# Ultrasonic Features of Uncommon Congenital Heterotopic Colon and Pancreas in the Neck: An Extremely Rare Case Report

**DOI:** 10.3389/fped.2021.655142

**Published:** 2021-05-28

**Authors:** Yingli Wei, Zhihao Pan, Xiaoling Kang, Cuiqing Huang, Dan Chen

**Affiliations:** ^1^Department of Ultrasound, Guangdong Women and Children Hospital, Guangzhou, China; ^2^Department of Pathology, Guangdong Women and Children Hospital, Guangzhou, China

**Keywords:** case report, color doppler flow imaging, congenital heterotopic colon, ultrasound, cyst, heterotopic pancreas

## Abstract

Congenital heterotopic colon and pancreas localized to the neck has not been reported. Herein, we describe an extremely uncommon case of congenital heterotopic colon and pancreas aberrantly presented within a cyst on the neck, and the thickened wall of the cyst on ultrasound may represent an important ultrasonic feature.

## Introduction

Ectopic gastrointestinal cysts are rare lesions that mainly occur in the digestive tract, including the esophagus, small intestine, pancreas, gallbladder and Meckel's diverticulum (1, 2). The lesions that occur in locations outside the digestive tract, such as the lips, larynx, submandibular gland, epiglottis and anterior neck, are even rarer (3, 4). To date, the exact etiology of ectopic gastrointestinal cysts remains unknown. It has been proposed that the cysts may arise from islands of the endoderm and originate from the lining of the primitive pores, and may be encapsulated at the embryonic age of 4 to 5 weeks when the entire gastrointestinal endoderm is undifferentiated (5).

An ectopic pancreas is an anatomically isolated pancreatic tissue outside of and separate to the main pancreas, with no anatomical or vascular connection to the normal pancreas (6). It is usually discovered by chance during surgery for another reason or at autopsy. In the general population, incidence rates range from 0.55 to 13.7% (autopsy) and 0.18–5.3% (surgery) (7), while neonatal patients are rarely reported. The pathogenesis of an ectopic pancreas has yet to be elucidated. It has been proposed that the occurrence is associated with certain abnormalities: the pancreatic primordium can be attached to or penetrate the wall of the colon, and this displacement can be transplanted abnormally with the growth and rotation of the intestine (8). An ectopic pancreas can occur at any location throughout the gastrointestinal tract, with the stomach and duodenum as the most common sites, followed by Meckel's diverticulum, the jejunum, and the ileum (9, 10). The abdominal cavity, lungs, and mediastinum are reported as less common locations (11–13). Pathological changes that occur in the ectopic pancreas include acute or chronic inflammation with fibrosis and pseudocyst formation, or even premalignant changes. To date, a lesion site in the neck has not been reported among cases with an ectopic pancreas. Herein, we report an extremely uncommon location of a congenital heterotopic colon and pancreas aberrantly presented in a cyst of the neck.

## Case Description

### Patient Description

A female infant was admitted to our hospital due to a cyst on the right side of the neck. The cyst was initially revealed when her mother underwent ultrasound examination at 29 weeks gestation. The cyst was ~3.0 × 1.8 × 2.2 cm in size, oval in shape, and had clear boundaries. Her mother did not have complications during pregnancy nor was there family history of heterotopic lesions.

During prenatal consultations, her mother learned about the risks and decided to continue the pregnancy. At 40 weeks of pregnancy, a cesarean section was performed to deliver the baby. Physical examinations showed that the birth weight was 3.1 kg and Apgar scoring was 9-10-10.

### Clinical Examinations and Initial Diagnosis

**Figure 1:**
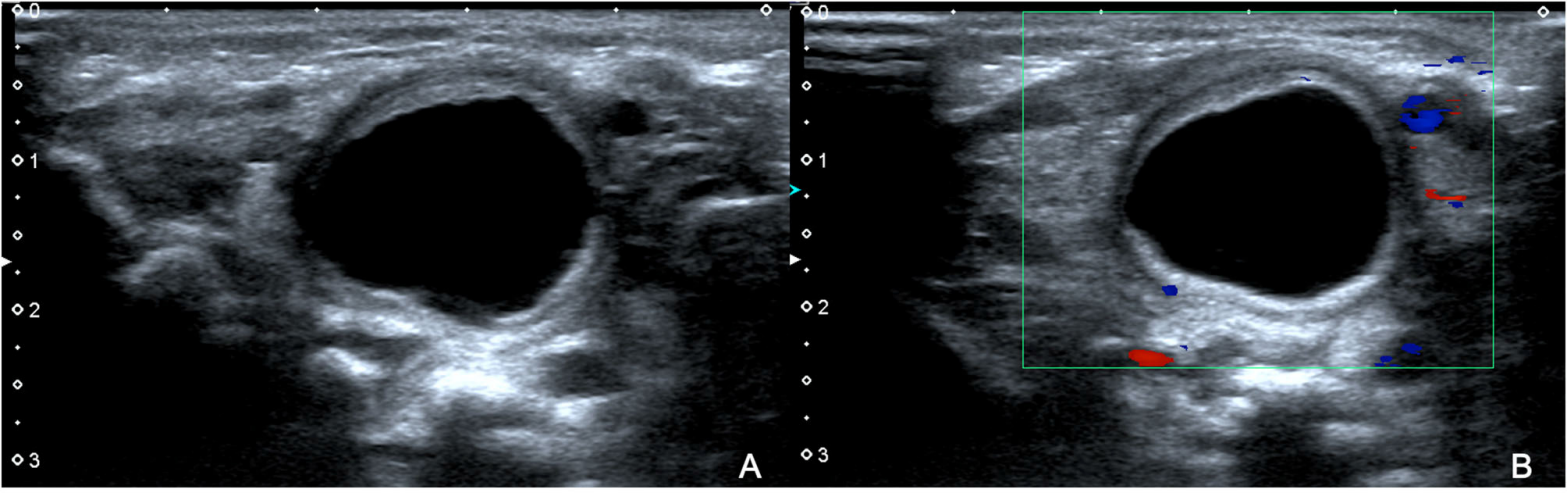
Ultrasound revealed a cyst on the right side of the neck in the patient at the age of 2 days. The cyst was 2.2 × 1.8 × 2.1 cm in size, and had a wall thickness of 0.2 cm **(A)**. Color Doppler flow imaging (CDFI) showed no obvious blood flow signal on the edge and wall of the capsule **(B)**.

**Figure 2:**
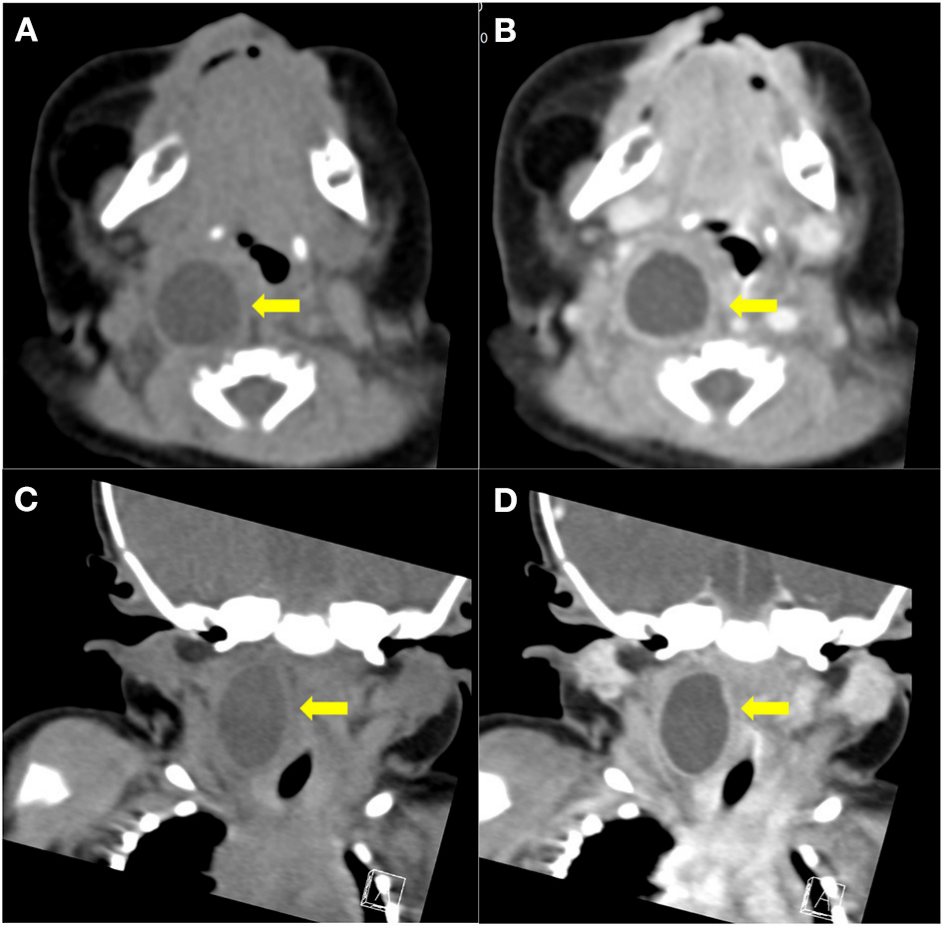
Cervical computed tomography in the patient at the age of 2 days. Coronal and sagittal imaging of cervical computed tomography (CT) revealed that the cyst (2.7 × 1.6 × 1.7 cm) was located in the parapharyngeal space close to the esophagus, and was causing mild compression of the trachea and cervical vessels **(A,C)**. No reinforcement was observed after contrast-enhanced CT scanning **(B,D)**.

Physical examinations revealed a soft neck, no mass on neck palpation, soft abdomen in the fasting state on abdominal palpation, no peristaltic wave, and normal stool. There were no respiratory or gastrointestinal symptoms in the patient. An ultrasound was performed and a cyst was detected on the right side of the neck, ~2.2 × 1.8 × 2.1 cm in size and with a wall thickness of 0.2 cm, clear boundaries, a regular shape, a translucent mass, and an unclear relationship with the esophagus. Color Doppler flow imaging (CDFI) showed no obvious blood flow signal on the edge and wall of the capsule (Figure 1). Computed tomography (CT) revealed that the cyst was located on the right side of the neck and the mass was compressing the trachea, right arteria cervicalis, and jugular vein (Figure 2). Laboratory test results were all within normal ranges. On the basis of the above examinations, a branchial fissure cyst was considered, and the initial diagnosis was made.

### Treatment, Final Diagnosis, and Prognosis

**Figure 3:**
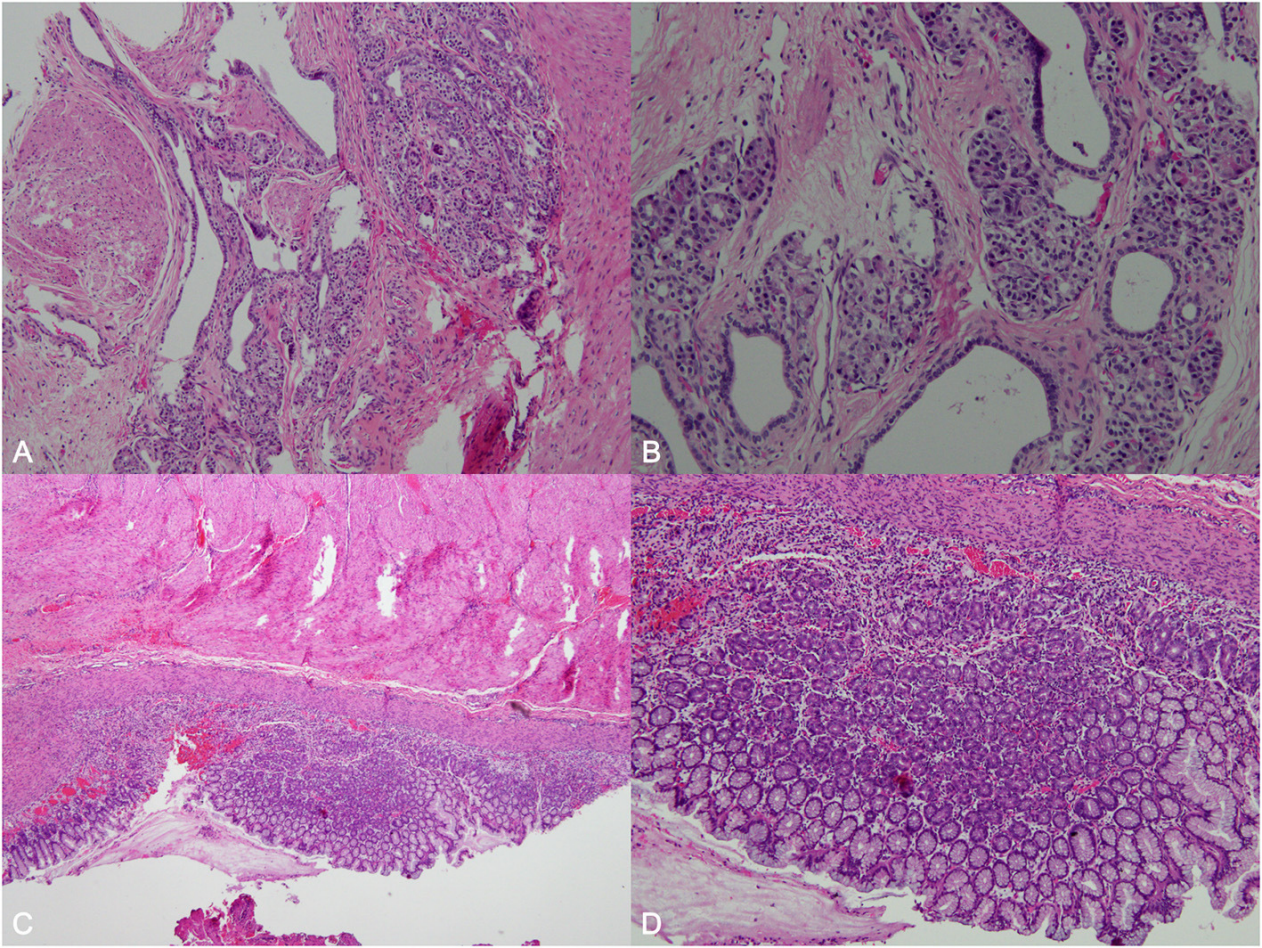
Histological examination of the resected specimen. Histological examination revealed that the cystic wall comprised of smooth muscle, and was lined with colonic mucosal tissue (hematoxylin and eosin [H&E]; × 100 magnification; **A,B**). The pancreatic components found consisted of acinar cells and ducts in the focal area (H&E; × 100; × 200; **C,D**).

The patient at the age of 3 days underwent surgical treatment for the cyst after her parent provided consent to the treatment procedures. The intraoperative findings showed that the mass was located close to the esophagus, trachea, and cervical spine. The wall of the capsule was thick, and the capsule fluid was light yellow and clear. Postoperative pathological examinations showed that the cystic wall consisted of smooth muscle and was lined with colonic mucosal tissue, and pancreatic components (acinar cells and ducts) were found in the focal area, and congenital colonic and pancreatic heterotopia were diagnosed and confirmed (Figure 3). Notably, the patient did not show any clinical signs of abnormalities during the 4-month follow-up period.

## Discussion

This case study illustrates an extremely rare case of a congenital heterotopic colon and pancreas tissue, with the neck as an uncommon site of the lesion in a 2-day-old female infant. The ultrasound findings in the patient indicated that the thickened wall of the cyst was an important ultrasonic feature of the congenital heterotopic colon and pancreas. Congenital heterotopic colon and pancreas is usually asymptomatic, which poses challenges for an early diagnosis and has even led to missed diagnoses in a proportion of patients. Therefore, the imaging characteristics may help to make an initial diagnosis. Furthermore, needle biopsy and histopathological examinations are required for a final diagnosis and confirmation. To the best of our knowledge, there has been no previous literature describing the ultrasound findings of a similar case, and as such this represents the first case report on this condition.

In this case, both the antenatal and postnatal ultrasound examinations revealed a cyst on the right side of the neck, and CT demonstrated that the cyst was close to the cervical vertebrae. An initial diagnosis of a fissure cyst was made based on the imaging results. The presence of the cyst caused oppression of ambient tissue, which was the primary reason that surgical resection was performed in the patient. To our surprise, the lesion was finally diagnosed and confirmed to be a congenital heterotopic colon and pancreas based on histopathological examinations of the resected tissue. Furthermore, it was noted that the acinar cells and ducts were similar to those found in the normal pancreas, suggesting that the cyst was congenital.

Ectopic gastrointestinal cysts are characterized by the presence of gastrointestinal smooth muscle, for which hypoechoic echo of the cystic wall is usually identified on the ultrasound image as an important ultrasonic feature of the disease. In this case, due to the lack of pancreatic tissue, it was difficult to identify the presence of an ectopic pancreas on the ultrasound image, and the final diagnosis only relied on pathological findings.

Common congenital cysts of the neck usually have the following characteristics: (1) clear boundary and regular shape; (2) translucent mass, dense floating spots, flocculent echo, separated light band or mesh echo, equal echo or slightly hyperechoic light mass when complicated with bleeding or infection; (3) thin wall; and (4) no blood flow signal on the edge of the cyst or on the cystic wall, but the blood flow signal can be displayed when complicated with bleeding or infection. Considering this case in combination with previous reports, the identification of cystic masses in the neck are as follows: (1) branchial fissure cyst: cervical side, mainly at the middle and lower 1/3 junction of the anterior edge of the sternocleidomastoid muscle; (2) thyrohyoid cyst: in the anterior middle of the neck, mostly between the hyoid bone and the thyroid gland; (3) lymphangioma: multiple compartmentalized cystic structure; or (4) congenital heterotopic colon and pancreas: thick cystic wall, characteristically similar to a gastrointestinal myometrial echo (14). The findings in this case may help to screen other cervical cysts. In addition, congenital heterotopic colon and pancreas tissue needs to be distinguished from gastrointestinal duplication cysts, since they present with similar ultrasonic features; however, the main identification points were location and relationship to the esophagus (15, 16). In light of potential complications due to cervical ectopic gastrointestinal cysts with a heterotopic pancreas (10, 17), and the adverse consequences including compression of the peripheral esophagus, trachea, blood vessels, asphyxia, and hypoxia, ultrasound can provide early initial diagnostic information according to characteristic sonography, and this is an important step toward implementing an effective intervention.

In conclusion, a congenital heterotopic colon and pancreas can aberrantly present within a cyst on the neck as an extremely rare location. Ultrasonic characteristics have been identified in this condition, which may help to distinguish other cervical cysts. When a congenital heterotopic colon and pancreas is suspected, CT and/or magnetic resonance imaging (MRI) scans should be performed for further assessment. A needle biopsy is necessary to make a definitive diagnosis, and surgical treatment is recommended to prevent potential complications.

## Data Availability Statement

The raw data supporting the conclusions of this article will be made available by the authors, without undue reservation.

## Ethics Statement

The studies involving human participants were reviewed and approved by Ethical Committee, Guangdong Women and Children Hospital. Written informed consent to participate in this study was provided by the participants' legal guardian/next of kin.

## Author Contributions

ZP and CH: drafting of the manuscript. DC and YW: critical revisions of the manuscript for important intellectual content. XK: clinical data collection, analysis, and interpretation. All authors contributed to the article and approved the submitted version.

## Conflict of Interest

The authors declare that the research was conducted in the absence of any commercial or financial relationships that could be construed as a potential conflict of interest.

## Abbreviations

CDFI: color Doppler flow imaging
CT: computed tomography
H&E: hematoxylin and eosin
MRI: magnetic resonance imaging
US: ultrasound.
